# Dosimetric evaluation of an ipsilateral intensity modulated radiotherapy beam arrangement for parotid malignancies

**DOI:** 10.2478/raon-2013-0010

**Published:** 2013-10-08

**Authors:** Eda Yirmibesoglu, David V. Fried, Mark Kostich, Julian Rosenman, William Shockley, Mark Weissler, Adam Zanation, Bhishamjit Chera

**Affiliations:** 1 Department of Radiation Oncology, Kocaeli University Faculty of Medicine, Kocaeli, Turkey; 2 Department of Radiation Oncology, University of North Carolina Hospitals, Chapel Hill, NC, USA; 3 Department of Otolaryngology/Head & Neck Surgery, University of North Carolina Hospitals, Chapel Hill, NC, USA; 4 Lineberger Comprehensive Cancer Center, University of North Carolina Hospitals, Chapel Hill, NC, USA

**Keywords:** intensity modulated radiotherapy, parotid, dosimetry

## Abstract

**Background:**

We conducted a dosimetric comparison of an ipsilateral beam arrangement for intensity modulated radiotherapy (IMRT) with off-axis beams.

**Patients and methods:**

Six patients who received post-operative radiotherapy (RT) for parotid malignancies were used in this dosimetric study. Four treatment plans were created for each CT data set (24 plans): 1) ipsilateral 4-field off-axis IMRT (4fld-OA), 2) conventional wedge pair (WP), 3) 7 field co-planar IMRT (7fld), and 4) ipsilateral co-planar 4-field quartet IMRT (4fld-CP). Dose, volume statistics for the planning target volumes (PTVs) and planning risk volumes (PRVs) were compared for the four treatment techniques.

**Results:**

Wedge pair plans inadequately covered the deep aspect of the PTV. The 7-field IMRT plans delivered the largest low dose volumes to normal tissues. Mean dose to the contralateral parotid was highest for 7 field IMRT. Mean dose to the contralateral submandibular gland was highest for 7 field IMRT and WP. 7 field IMRT plans had the highest dose to the oral cavity. The mean doses to the brainstem, spinal cord, ipsilateral temporal lobe, cerrebellum and ipsilateral cochlea were similar among the four techniques.

**Conclusions:**

For postoperative treatment of the parotid bed, 4-field ipsilateral IMRT techniques provided excellent coverage while maximally sparing the contralateral parotid gland and submandibular gland.

## Introduction

The standard of care treatment of parotid gland malignancies is surgery followed by postoperative radiotherapy (RT) when indicated. Postoperative RT has been shown to improve loco-regional control and is generally recommend for tumors with high-risk features such as high grade, positive or close margins, lymph node metastases, or tumor recurrence.^1^ The post-surgical parotid bed and cranial portions of level II are the primary targets and levels I – III of the ipsilateral neck are treated electively if the neck has not been dissected and adjuvantly if there are ≥ 2 positive nodes or node(s) and/or node(s) with extracapsular extension.

Historically, a wedge pair technique was used to treat the post-surgical parotid bed. If there were indications for treatment of the neck, an isocentrically matched low anterior neck field was added. The ipsilateral wedge pair beam arrangement creates a “pie” shaped dose distribution while sparing the contralateral parotid and submandibular gland. The potential shortcoming of the wedge pair technique is suboptimal coverage of the deep aspects of the parotid bed (*e.g*. deep lobe of the parotid). Contemporarily intensity modulated radiotherapy (IMRT) is the most frequently utilized radiation treatment technique for head and neck cancer and is frequently used for the postoperative treatment of parotid and other malignancies.^2^ The potential benefits of IMRT include improved normal tissue sparing and target coverage through highly conformal dose distributions with steep dose gradients. Typically, IMRT is delivered with 7 to 9 axial beams equidistantly spaced along the transverse axis. The use of multiple co-planar beams (as compared to ipsilateral beams) will result increased dose to the contralateral parotid and submandibular glands which may cause significant xerostomia. However, multiple beams from various angles provide more degrees of freedom and thus improve the dose to the deep aspects of the post-surgical parotid bed.

At our institution patients receiving postoperative radiotherapy for parotid malignancies are treated with IMRT using an ipsilateraloff-axisquartet beam arrangement. This technique maximally spares the contralateral parotid and submandibular glands and provides excellent coverage of the deep aspects of the parotid bed. Hence it combines the benefits of the wedge pair and the 7 field coplanar IMRT techniques. We here-in describe our 4-fieldoff-axisipsilateral IMRT technique and present a dosimetric comparison of this technique to conventional wedge pair, 4-field co-planar IMRT, and 7 field co-planar IMRT plans.

## Patients and methods

We obtained approval from our institutional IRB for this study (IRB# 09-2146). The current dosimetric study used computed tomography (CT) simulation data sets from 6 patients who were treated with postoperative radiotherapy for parotid malignancies. All of these patients had elective neck dissections and were pathologically node negative. Thus none of these patients received neck irradiation. 5 out of 6 of these patients had already received postoperative radiation treatment using the 4-field off-axis ipsilateral IMRT technique. One patient was treated with a conventional wedge pair technique prior to our institutions adoption of the 4-field ipsilateral IMRT technique.

### CT Simulation

Patients were placed in the supine position and the head and neck were immobilized with a customized AquaPlast mask (WFR/Aquaplast Corp. and Qfix Systems, Avondale, PA). The neck was extended and the shoulders were relaxed downward with gentle traction. CT images were obtained with a Philips Brilliance Big Bore, 16-slice CT scanner (Amsterdam, Netherlands). The image slice thickness was 3 mm, and patients were scanned from the vertex to the below the clavicles. Intravenous contrast was not used.

### Volume definition

Target and organ at risk volumes (OARs) were delineated using PLUNC. The clinical and planning target volumes (CTV, PTV) that were delineated at the time of their initial treatment planning were used for this dosimetric study. Typically the CTV encompassed the post-surgical parotid bed, adjacent parapharyngeal space, and course of the facial nerve to the styloidmastoid foramen. None of the 6 patients in this study had a positive proximal facial nerve margin and thus neither the CTV nor the PTV encompassed the course of the facial nerve proximal to the styloidmastoid foramen (*i.e.* inner ear and/or brainstem). The PTV was created by uniformaly expanding the CTV by 3 mm. The PTV and CTV were subtracted 4 mm within the skin. The following OARs were contoured: contralateral parotid gland, contralateral submandibular gland, ipsilateral cochlea, contralateral cochlea, ipsilateral temporal lobe, brainstem, cerebellum, and cervical spinal cord. OARs were uniformly expanded 3 mm to create planning risk volumes (PRVs).

### Treatment planning and prescription dose

Four treatment plans were created for each CT data set (24 plans): 1) conventional wedge pair, 2) 7-field co-planar IMRT, 3) 4-fieldoff-axis IMRT and 4) 4-field co-planar IMRT. 70 Gy was prescribed to the PTV. 70 Gy was chosen because it represents the maximum dose prescribed for this treatment.

For the conventional wedge pair two oblique fields (approximately with 90^°^ angles) were used. The beam angles for the oblique fields were chosen to exclude the contralateral parotid and submandibular glands and provide adequate coverage of the PTV. Furthermore one of the oblique beams excluded the brainstem and spinal cord. Wedges were used for both oblique fields and weighted to provide acceptable coverage of the PTV. By convention and in accordance with historical standards the wedge pair plans were normalized such that 95% of the PTV received 95% of the dose. Normalization to 100% would result in excessive heterogeneity (≥120%) that would be clinical unacceptable.

**FIGURE 1.**
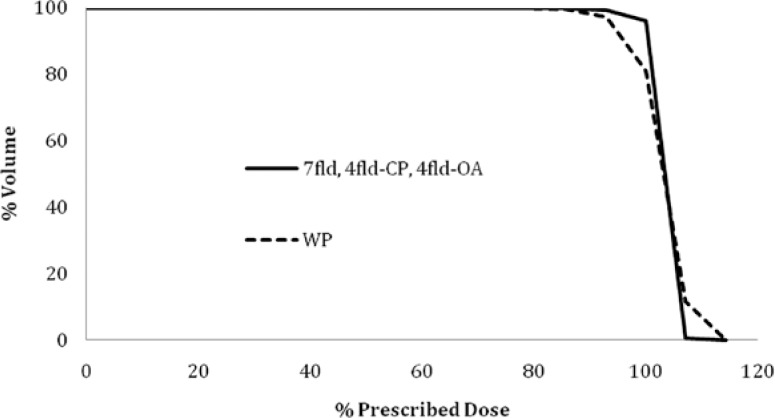
Three-dimensional representation of the orientation of the 4 beams used in the 4-field off-axis IMRT plans for a target on the right side of a patient. A45R-45I = 45 degree right anterior oblique beam with a 45 degree inferior Table kick; A45R-45S = 45 degree right anterior oblique beam with a 45 degree superior Table kick; P45R-45I = 45 degree right posterior oblique beam with a 45 degree inferior Table kick; P45R-45S =45 degree right posterior oblique beam with a 45 degree superior Table kick.

For the 7-field co-planar IMRT seven equally spaced beams (approximately every 52^°^) were isocentrically centered on the PTV. The field and table angles for the 4-field off axis IMRT plan were: A45R-45I, A45R-45S, P45R-45I, P45R-45S (right side) and A45L-45S, A45L-45I, P45L-45S, P45L-45I (left side) (Figure 1). For example A45R-45I translates into a 45 degree right anterior oblique beam with a 45 degree inferior tablekick. These beam angles were selected to exclude the eye, shoulder, and contralateral parotid and submandibular glands from the beams. Depending on a patient’s anatomy the beam angles may require minor adjustments to exclude the eye and shoulder. Another 4-field coplanar IMRT plan was created for comparison purposes according to the 4-field class solution published by Nutting *et al*. The 4-field coplanar IMRT plan consisted of paired ipsilateral co-planar anterior andposterior oblique beams with the following angles: 15, 45, 145, and 170° form the anterior plane.^3^

The IMRT treatment planning process was similar for both the 7-field and 4-field IMRT plans.^4^ Dose objectives were iteratively selected for the PTV and PRVs to meet pre-defined dose constraints. Ghost structures were also used to improve dose conformity around the PTV and avoidance of PRVs. 70 Gy was prescribed to the PTV. The PTV dose constraints were: 95% of the PTV receives 100% of the prescription dose, 99% of the PTV receives 93% of the prescription dose, and <20% of the PTV receives 110% of the prescription dose. Because the contralateral parotid and submandibular glands are in the beam path of several beams in the 7-field IMRT, these PRVs were included in the IMRT optimization for the 7-field treatment plans. The dose to the contralateral parotid and submandibular gland was minimized as much as allowable while meeting the dose constraints for the PTV. It was unnecessary to optimize the 4-field IMRT plan for the contralateral parotid and submandibular gland, because the beams excluded the contralateral parotid and submandibular gland.

### Plan evaluation and comparison

Dose, volume statistics were collected for PTV, CTV, and PRVs for all 24 plans. The maximum dose and the minimum dose weredefined as the dose received by 0.1cc of the defined structure. Volume of PTV and non-specified normal tissues outside of the PTVreceiving 70Gy, 66.5Gy (*i.e*. 95% of prescription dose), 50Gy, 35Gy and 14Gy were recorded to assess the conformality of each plan. The mean values and standard deviations were calculated for reported dose, volume statistics. Dose volume histograms (DVH) were created for PTV, and PRVs.

## Results

### Dose to CTV and PTV

**FIGURE 2.**
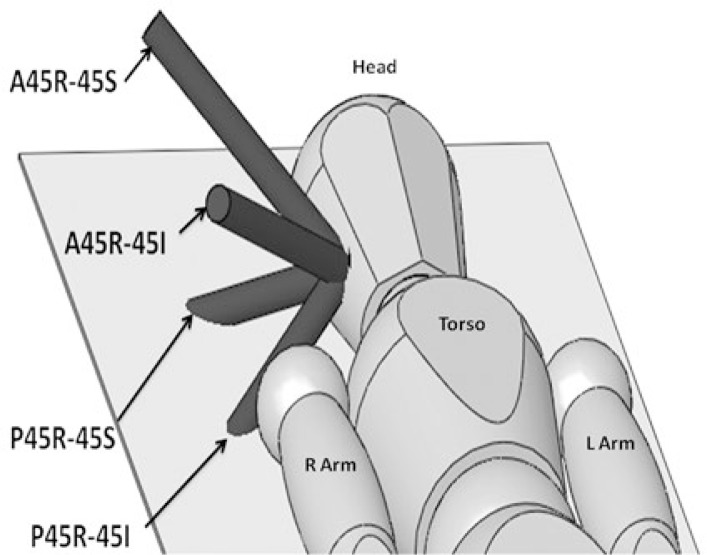
Dose volume histogram of the Planning Target Volume (PTV) for wedge pair (WP), 7-field IMRT (7fld), 4-field co-planar (4fld-CP), and 4-field off-axis (4fld-OA) IMRT plans.

**FIGURE 3.**
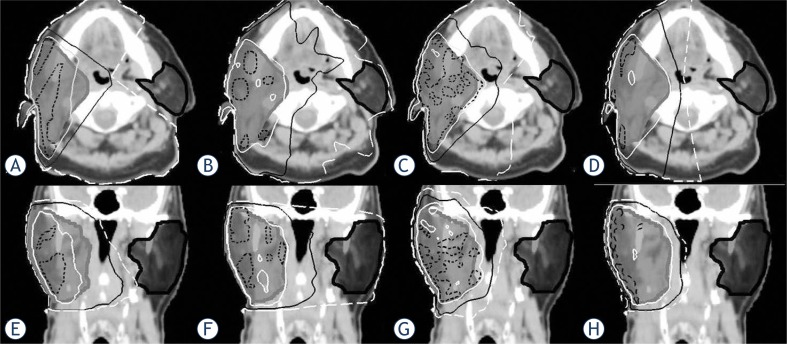
Transverse and coronal view of computed tomography images with isodose distributions at the isocenter level in a representative patient with left sided disease. (A, E) wedge pair; (B, F) 7-field IMRT; (C, G) 4-field IMRToff-axis; (D,H) 4-field IMRT co-planar. The isodoses are 105% (dashed black), 100% (white), 50% (black) and 25% (dashed white) of the prescribed dose (70Gy).

Dose volume data for the PTV are listed in Table 1 and related dose volume histograms in Figure 2. Representative isodose distributions for the four treatment plans are depicted in Figure 3. The PTV coverage was similar for both the 7 field and 4 field IMRT plans. As expected, the medial aspect of the PTV (*i.e*. deep aspect of the post-surgical parotid bed) was inadequately covered with the wedge pair plans (Figure 3, A & E). Dose statistics regarding the conformality of the high dose (*i.e.* 100% and 95% of the prescription dose) and the integral dose are shown in Table 2. The wedge pair had the worst coverage of the PTV in terms of dose covered by 100% and 95% of the prescription. The wedge pair and 7-field IMRT plans had the least high dose delivered to normal tissues, and the four field plans had much less low dose (*i.e*. integral dose) delivered to normal tissues.

### Dose to PRVs

**FIGURE 4.**
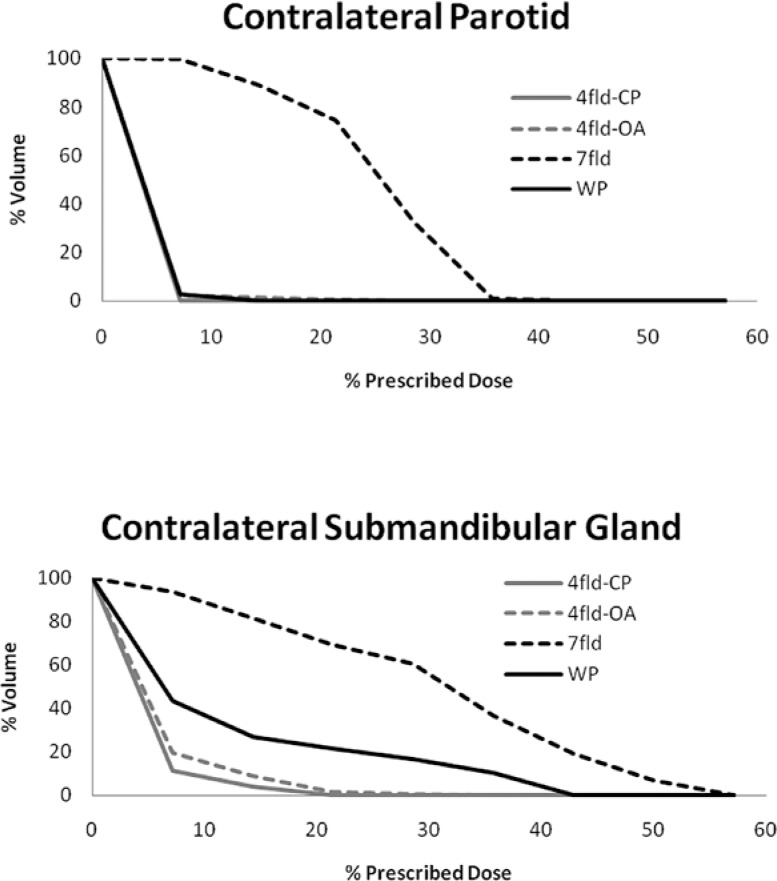
Dose volume histograms of the contralateral parotid gland and contralateral submandibular gland for for wedge pair (WP), 7-field IMRT (7fld), 4-field co-plananr (4fld-CP), and 4-field off-axis (4fld-OA) IMRT plans.

**FIGURE 5.**
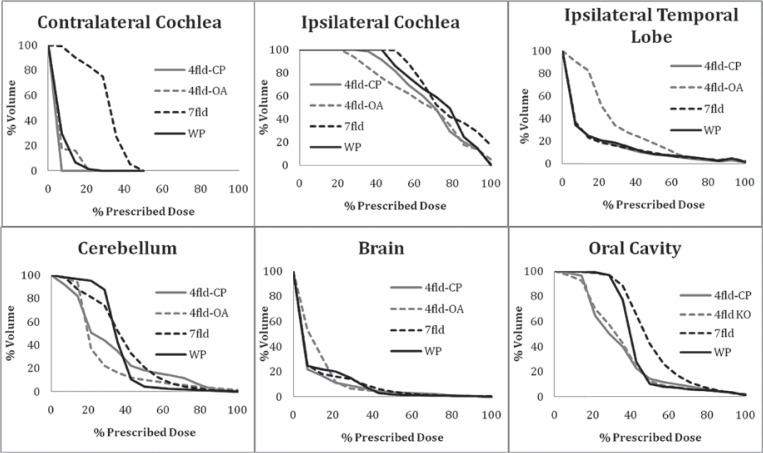
Dose volume histograms of the ipsilateral cochlea, contralateral cochlea, ipsilateral temporal lobe and cerebellum for wedge pair (WP), 7-field IMRT (7fld), 4-field co-plananr (4fld-CP), and 4-field off-axis (4fld-OA) IMRT plans.

Dose volume data for the PRVs can be found in Table 3 and Figure 4 and 5. Due to the ipsilateral beam arrangement, the mean doses for the contralateral parotid gland, submandibular gland and cochleare lower in the wedge pair plan and 4-field IMRT plans. The mean dose and maximum dose to the ipsilateral cochlea are similar for all four plans. There is no clinically significant difference in the maximum dose and the volume receiving ≥60Gyto the brain, ipsilateral temporal lobe, and cerebellum. The maximum dose to the brainstem and cervical spinal cord were similar in all techniques. The mean dose to the oral cavity was highest for the 7 field IMRT plan and least for the 4 field IMRT plans.

## Discussion

We performed a dosimetric study comparing our institutional specific ipsilateral 4-field off-axis IMRT treatment technique with conventional wedge pair, 7-field co-planar IMRT, and 4-field co-planar IMRT plans for the postoperative treatment of parotid gland malignancies. As expected the ipsilateral 4-Field IMRT techniques spared the contralateral parotid gland and submandibular gland as well as the wedge pair technique and provided PTV coverage similar to the co-planar 7-field IMRT technique. Furthermore, the deep/medial aspects of the PTV and CTV were underdosed with the wedge pair technique. Thus the ipsilateral 4-field IMRT techniques combine the benefits of the wedge pair and co-planar 7 field IMRT plans. Thus, the ipsilateral 4-field IMRT technique may be the preferred method for irradiating the post-surgical parotid bed. The two 4-field IMRT techniques were very similar to one another. However there is an increase in low dose (*i.e*. <10Gy IDL) to the brain and neck and increased complexity because of the use of table kicks with our institution specific 4-field off-axis IMRT plan.

Historically, the conventional wedge pair technique has been predominantly used for the postoperative treatment of parotid malignancies.^1^ Other ipsilateral techniques, such as mixed photon electron beams, have been used and evaluated. Yaparpalvi *et al.* conducted a study concerning comparison of unilateral radiotherapy techniques for postoperative parotid gland tumors based on dose distribution and DVHs, the concluded that the ipsilateral wedge pair technique was the optimal unilateral treatment techniques.^5^

IMRT treatment planning is extensively used for the treatment of head and neck cancers. IMRT produces highly conformal dose distribution that can reduce the dose to normal tissue structures. Specifically, IMRT has been observed to improve reduce the severity of xerostomia through the sparing of the contralateral parotid gland.^6^ The most common beam arrangement used for IMRT planning is 7 to 9 equally spaced co-planar beams. When ipsilateral RT is possible (*e.g*. parotid malignancies) the 7 to 9 equidistant beam arrangement is not optimal. More beams increase the conformity of the high dose distribution at the cost of increasing the dose to contralateral normal tissues. In addition to the presented data, others have conducted dosimetric studies of IMRT for parotid malignancies. Nutting *et al*. and Rowbottom *et al*. reported that IMRT with seven to nine fields reduced the dose to most normal tissues compared to conventional wedge pair, but the dose to contralateral OARs was increased.^3^,^7^ Furthermore Nutting *et al*. also evaluated 3- and 4-field off-axis IMRT beam arrangements but these plans increased the PTV dose inhomogeneity, and increased the dose to the brain.^3^ They observed 17.6 ccs of brain received > 54 Gy in their 4-field off-axis plan *vs*. 1.9 cc in their coplanar 4-field IMRT class solution plan and 2 cc in their 7-field co-planar IMRT plan. They did not detail the beam arrangements for these off-axis IMRT plans.^3^ In comparison we did not observe a significant increase PTV dose heterogeneity in our 4-field off-axis IMRT technique. We did observe an increase in low dose to the brain (Figure 5) for the 4-field off-axis technique, however, the volume of brain, ipsilateral temporal lobe, and cerebellum receiving > 60 Gy was similar for the 7-field co-planar IMRT, 4-field off-axis, and 4-field coplanar IMRT plans.

The contralateral lymphatics are rarely at risk in parotid malignancies; the possible exception being large volume ipsilateral nodal disease potentially causing aberrant lymphatic flow to the contralateral neck. Thus, the contralateral side may be completely spared from RT. The most efficient method for minimizing radiation dose to the contralateral side is careful beam selection. “Beam optimization” is a primary first step in IMRT treatment planning and must be done by the dosimetrist/physician/physicist. When using IMRT, the standard 7 to 9 field equidistant field arrangement used for the majority of head and neck cancer IMRT is not the optimal beam arrangement for post-operative RT of parotid malignancies. This beam arrangement substantially increases the dose delivered to the contralateral normal tissues, especially the major salivary glands. Bragg *et al*. reported that a five field beam arrangement was optimal; however the resultant mean dose the contralateral parotid was 10 to 11 Gy.^8^ We observed the contralateral parotid and submandibular gland radiation dose in the 7-field IMRT plan to be below the commonly accepted tolerance dose (*i.e*. mean dose < 26 Gy and mean dose < 35 Gy), however the dose to these structures was substantially lower for the 4-field IMRT plans (Table 3, 17 to 21 Gy *vs*. 1 to 2 Gy). In fact, the dose to the contralateral major salivary glands was similar for the wedge pair and 4-field IMRT plans (Table 3). It is reasonable to rationalize that 17 to 20 Gy to the contralateral salivary glands will impair salivary production. Previous studies have reported that mean doses < 10-15 Gy to the salivary gland resulted in minimal reduction in function and impairment in salivary function gradually increased at radiation doses of 20–40 Gy.^9^,^10^ Furthermore, the recent QUANTEC review, noted that mean doses < 10 Gy to the parotid gland resulted in better function.^11^

The 4-field off-axis and co-planar IMRT plans both provide adequate coverage of the PTV/CTV, have a similar conformality of the high dose region, and maximally spare the contralateral major salivary glands. However, the integral brain dose is higher in the 4-field off-axis planar plan. The 4-field off-axis plan is also more complicated because “Table kicks” are required. Furthermore if the ipsilateral neck requires RT, it would be difficult to match a low anterior neck field to the 4-field off-axis plan. Thus the 4-field co-planar IMRT technique published by Nutting *et al*. is the better 4-field technique. Since conduction of this dosimetric study, we at UNC-CH have transitioned from the 4-field off-axis technique to using the 4-field coplanar technique.

## Conclusions

For postoperative treatment of the parotid bed, the ipsilateral 4-field off-axis or co-planar IMRT techniques provide excellent target coverage (specifically the deep/medial aspect of the parotid bed) while maximally sparing the contralateral parotid and submandibular glands. Should cervical nodes require treatment, it may be difficult to match a low anterior neck field to the 4-field off-axis technique. Furthermore the 4-field off-axis technique is more complex because of the use of table kicks. The 4-field co-planar IMRT technique is preferable for the postoperative treatment of the parotid bed.

## Figures and Tables

**TABLE 1.** Dose Volume Statistics for Planning Target Volume (PTV) and Clinical Taget Volume (CTV)

	**Wedge pair**		**7 field co-planar IMRT**		**4-field off-axis**		**4-field co-planar**	

	**Mean**	**SD**	**Mean**	**SD**	**Mean**	**SD**	**Mean**	**SD**
**PTV**								
Mean (Gy)	71.6	0.65	71.8	0.56	71.8	0.61	71.5	0.36
Max dose (Gy)	77.2	0.71	75.5	0.98	75.9	1.3	75.6	0.67
Min dose(Gy)	51.2	5.9	65.2	2.8	57.3	8.9	58.1	6.7
V95 (%)	95.4	0.45	99.9	0.15	99.4	0.49	99.1	0.72
V105 (%)	31.3	10.7	14.3	14.7	16.8	13.6	8.8	5.2
V110 (%)	1.23	2.88	0	0	0.01	0	0	0
**CTV**								
Mean (Gy)	71.8	0.92	71.9	0.58	71.9	0.60	70.6	0.90
Max dose (Gy)	75.5	0.60	75.5	0.92	75.8	1.2	75.5	0.60
Min dose(Gy)	59.1	8.8	66.5	2.3	59.5	7.1	59.1	8.8
V95 (%)	97.2	3.7	99.9	0.2	99.4	0.5	97.2	3.7
V105 (%)	4.75	4.3	15.3	15.4	17.9	14.3	4.8	4.3
V110 (%)	1.1	2.7	0	0	0	0	0	0

IMRT = Intensity modulated radiation therapy; SD = standard deviation; PTV = planning target volume; Max = maximum dose received by 0.1cc; Min = minimum dose received by 0.1cc; V95(%), V105 (%), V100, V110(%) = percentage of volume receiving 95%, 105%, 100%, and 110% of prescribed dose, respectively

**TABLE 2.** Conformality of the high dose region and the integral dose

**Isodose Volume**	**Wedge pair**			**7 field co-planar IMRT**			**4-field off-axis IMRT**			**4-field co-planar IMRT**		

	**Mean (cc)**	**% PTV**	**SD**	**Mean (cc)**	**% PTV**	**SD**	**Mean (cc)**	**% PTV**	**SD**	**Mean (cc)**	**% PTV**	**SD**
**70 Gy to PTV**	157	81	42	188	97	52	189	97	52	191	98	53
**70 Gy to NT**	71	-	15	73	-	22	101	-	33	116	-	19
**66.5 Gy to PTV**	185	95	52	193	100	53	193	100	53	192	100	53
**66.5 Gy to NT**	127	-	16	129	-	23	144	-	30	153	-	19
50 Gy to NT^*^	243	-	43	292	-	47	276	-	41	360	-	46
35 Gy to NT^*^	342	-	48	597	-	84	424	-	69	484	-	59
14 Gy to NT^*^	1647	-	281	1724	-	320	1205	-	159	1070	-	104

Volume of Normal Tissue (NT) and Planning Target Volume (PTV) receiving various doses. Normal tissue represents all of normal tissue outside of the PTV (i.e. Skin minus PTV). The intergral dose (14 to 50 Gy) was higher in the 7 field IMRT plan. However, the volume of NT receiving 95% of the prescription dose (i.e. 66.5 Gy) was smallest for the 7 field IMRT plan. IMRT = intensity modulated radiotherapy.

* Since plans were prescribed 70 Gy at the 95%-100% isodose line, all PTV’s received 100% coverage of the 50 Gy, 24 Gy, and 14Gy.

**TABLE 3.** Dose volume statistics for planning risk volumes

	**Wedge pair**		**7 field co-planar IMRT**		**4-field off-axis IMRT**		**4-field co-planar IMRT**	

	**Mean**	**SD**	**Mean**	**SD**	**Mean**	**SD**	**Mean**	**SD**
**Mean Dose Contralateral Parotid (Gy)**	2.6	0.54	17.4	2.0	1.3	0.84	0.91	0.12
**Mean Dose Contralateral Submandibular (Gy)**	8.5	4.5	20.5	6.8	3.6	1.5	2.6	0.86
**Ipsilateral Cochlea**								
**Mean (Gy)**	50.8	9.0	54	12.2	44.9	14.6	47.7	9.7
**Max dose (Gy)**	60.6	8.6	59.8	11.8	56.7	11.5	58.4	9.2
**Contralateral Cochlea**								
**Mean (Gy)**	4.8	1.7	21	6.2	3.5	4.6	1.6	0.2
**Max dose (Gy)**	7.4	3.9	24.5	5.4	4.6	4.9	2.3	0.3
**Brain**								
**Max dose (Gy)**	71.7	3.0	71.4	2.6	72.6	1.9	71.6	2.0
**Volume ≥60 Gy (cc)**	8.1	6.3	10.5	14.0	11.5	13.0	10.6	10.3
**Ipsilateral Temporal Lobe**								
**Max dose (Gy)**	68.3	3.4	69.8	3.7	67.7	5.4	68.7	4.7
**Volume ≥60 Gy (cc)**	4.6	4.7	4.8	8.6	3.4	5.3	3.0	4.0
**Cerebellum**								
**Max dose (Gy)**	68.5	4.2	68.4	4.8	68.8	7.8	69.6	3.4
**Volume ≥60 Gy (cc)**	1.5	1.5	2.7	2.8	5.5	5.4	4.2	3.7
**Max Dose Brain Stem(Gy)**	38.6	8.3	45.1	8.7	32.8	7.8	36.3	5.7
**Max Dose Spinal Cord(Gy)**	40.3	6.5	45.9	7.0	39.8	4.9	43.0	5.6
**Oral Cavity (Gy)**	30.1	2.8	35.8	4.5	24.1	4.8	23.6	2.7

PRVs= planning risk volumes; IMRT= Intensity modulated radiation therapy; SD= standard deviation; Max= maximum (dose received by 0.1cc); Volume ≥60Gy= volume receiving ≥60Gy.

## References

[1] Garden AS, el-Naggar AK, Morrison WH, Callender DL, Ang KK, Peters LJ (1997). Postoperative radiotherapy for malignant tumors of the parotid gland. Int J Radiat Oncol Biol Phys.

[2] Bailey DW, Kumaraswamy L, Podgorsak MB (2010). A fully electronic intensity-modulated radiation therapy quality assurance (IMRT QA) process implemented in a network comprised of independent treatment planning, record and verify, and delivery systems. Radiol Oncol.

[3] Nutting CM, Rowbottom CG, Cosgrove VP, Henk JM, Dearnaley DP, Robinson MH (2001). Optimisation of radiotherapy for carcinoma of the parotid gland: a comparison of conventional, three-dimensional conformal, and intensity-modulated techniques. Radiother Oncol.

[4] Chang SX, Cullip TJ, Rosenman JG, Halvorsen PH, Tepper JE (2002). Dose optimization via index-dose gradient minimization. Med Phys.

[5] Yaparpalvi R, Fontenla DP, Tyerech SK, Boselli LR, Beitler JJ (1998). Parotid gland tumors: a comparison of postoperative radiotherapy techniques using three dimensional (3D) dose distributions and dose-volume histograms (DVHS). Int J Radiat Oncol Biol Phys.

[6] Nutting CM, Morden JP, Harrington KJ, Urbano TG, Bhide SA, Clark C (2011). Parotid-sparing intensity modulated versus conventional radiotherapy in head and neck cancer (PARSPORT): a phase 3 multicentre randomised controlled trial. Lancet Oncol.

[7] Rowbottom CG, Nutting CM, Webb S (2001). Beam-orientation optimization of intensity-modulated radiotherapy: clinical application to parotid gland tumours. Radiother Oncol.

[8] Bragg CM, Conway J, Robinson MH (2002). The role of intensity-modulated radiotherapy in the treatment of parotid tumors. Int J Radiat Oncol Biol Phys.

[9] Chao KS, Deasy JO, Markman J, Haynie J, Perez CA, Purdy JA (2001). A prospective study of salivary function sparing in patients with head-and-neck cancers receiving intensity-modulated or three-dimensional radiation therapy: initial results. Int J Radiat Oncol Biol Phys.

[10] Blanco AI, Chao KS, El Naqa I, Franklin GE, Zakarian K, Vicic M (2005). Dose-volume modeling of salivary function in patients with head-and-neck cancer receiving radiotherapy. Int J Radiat Oncol Biol Phys.

[11] Deasy JO, Moiseenko V, Marks L, Chao KS, Nam J, Eisbruch A (2010). Radiotherapy dose-volume effects on salivary gland function. Int J Radiat Oncol Biol Phys.

